# Ceph-X: development and evaluation of 2D cephalometric system

**DOI:** 10.1186/s12859-016-1370-5

**Published:** 2016-12-22

**Authors:** Mogeeb Ahmed Ahmed Mosleh, Mohd Sapiyan Baba, Sorayya Malek, Rasheed A. Almaktari

**Affiliations:** 1 Software Engineering Department, Faculty of Engineering & Information Technology, Taiz University, 6169 Taiz, Yemen; 2 Institute of Biological Sciences, Faculty of Science, University of Malaya, 50603 Kuala Lumpur, Malaysia; 3 Faculty of Dentistry, Orthodontic Department, University of Malaya, 50603 Kuala Lumpur, Malaysia

**Keywords:** Computer-aided biomedical image, Automated cephalometric, Digital image processing, Evaluation cephalometric system

## Abstract

**Background:**

Cephalometric analysis and measurements of skull parameters using X-Ray images plays an important role in predicating and monitoring orthodontic treatment. Manual analysis and measurements of cephalometric is considered tedious, time consuming, and subjected to human errors. Several cephalometric systems have been developed to automate the cephalometric procedure; however, no clear insights have been reported about reliability, performance, and usability of those systems. This study utilizes some techniques to evaluate reliability, performance, and usability metric using SUS methods of the developed cephalometric system which has not been reported in previous studies.

**Methods:**

In this study a novel system named Ceph-X is developed to computerize the manual tasks of orthodontics during cephalometric measurements. Ceph-X is developed by using image processing techniques with three main models: enhancements X-ray image model, locating landmark model, and computation model. Ceph-X was then evaluated by using X-ray images of 30 subjects (male and female) obtained from University of Malaya hospital. Three orthodontics specialists were involved in the evaluation of accuracy to avoid intra examiner error, and performance for Ceph-X, and 20 orthodontics specialists were involved in the evaluation of the usability, and user satisfaction for Ceph-X by using the SUS approach.

**Results:**

Statistical analysis for the comparison between the manual and automatic cephalometric approaches showed that Ceph-X achieved a great accuracy approximately 96.6%, with an acceptable errors variation approximately less than 0.5 mm, and 1°. Results showed that Ceph-X increased the specialist performance, and minimized the processing time to obtain cephalometric measurements of human skull. Furthermore, SUS analysis approach showed that Ceph-X has an excellent usability user’s feedback.

**Conclusions:**

The Ceph-X has proved its reliability, performance, and usability to be used by orthodontists for the analysis, diagnosis, and treatment of cephalometric.

## Background

Cephalometric is a compound latin word includes two distinct terms: *cephalo* (the head), and *metrics* (measurements) [1]. Thus, cephalometry is the art of the human head measurements which used to evaluate craniofacial growth. Skull radiographs is involved widely to measure the human head dimensions since several years ago [2].

Skull relationship can be evaluated by using cephalometric techniques for both horizontally and vertically of five major features through linear and angular measurements. These features are the skeletal maxilla, the skeletal mandible, the cranium and cranial base, the maxillary dentition and the mandibular dentition [3].

**Fig. 1:**
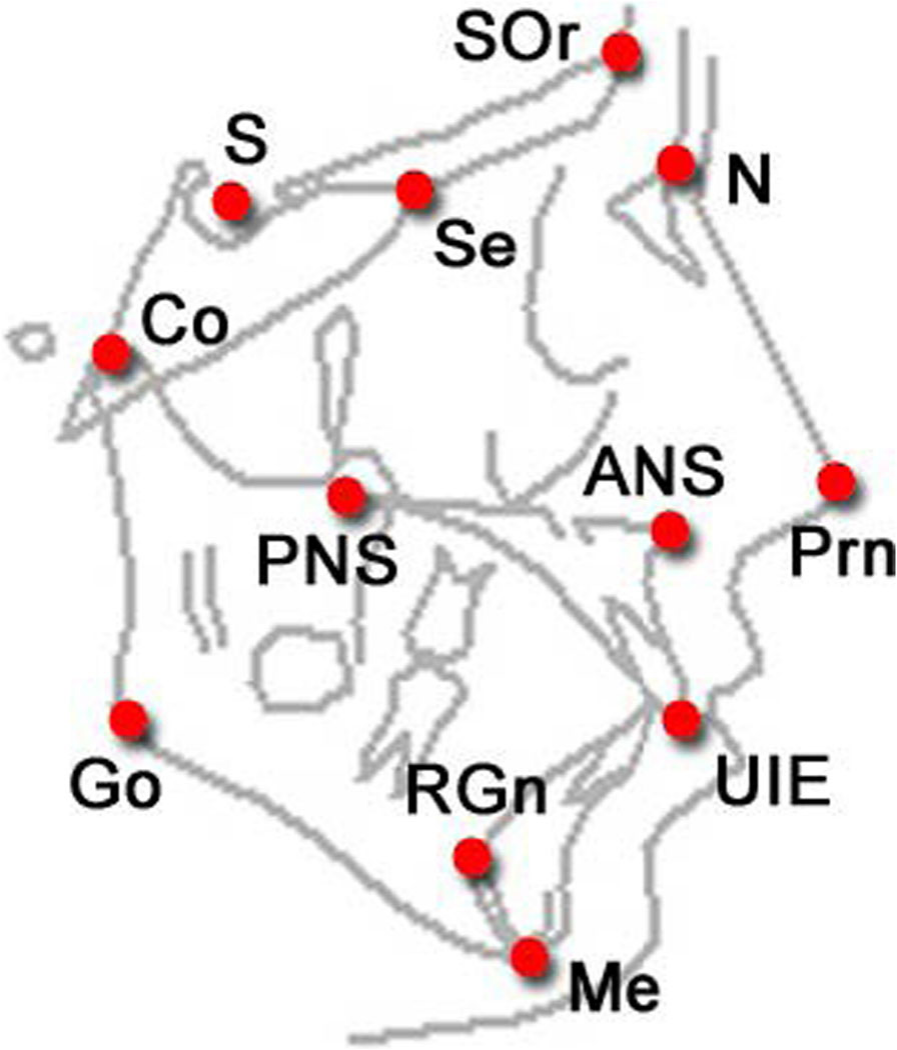
Cephalometric Landmark Points

Maxillofacial surgery, and orthodontics uses X-ray images to mark specific point on skull to obtain the various angular and linear parameters [4]. Those points called cephalometric landmark which identified as set of feature in both hard and soft tissue of the skull. Landmarks are employed to measure the cephalometric components as distance in millimetres, and angles in degree [4]. Landmarks are common anatomical points in human skeleton as represented in Fig. 1. There are nearly 20 to 30 landmarks on the human skull which used widely in cephalometric measurement [5].

Orthodontics used several techniques for cephalometric analysis and measurements by using angular and linear measurements. Angular analysis is used to establish the relations between the individual sections of the skull, while the linear analysis is used to obtain the distance between two reference points in the skull [6]. Orthodontics usually uses their experiences to locate cephalometric landmarks manually on radiographic images. Unfortunately, the manual process is exposed to human errors such as projection errors during the conversion between the 3-D image and the 2-D image [7], X-ray film errors due to the clarity and device resolution [8], and measurements errors due to the human eyes limitation, pencils thickness, and unskilful hands [7]. In addition, the conventional method is also considered tedious and time consuming process taking on average 15 to 20 min from expert specialist to handle each individual case [9, 10].

Computerizing cephalometric have been employed to solve the previous issues, and to offer numerous advantages such as reduce the efforts and times of orthodontic, X-ray enhancement, consistent measurements, pre-surgical simulation, obtain more accurate and reliable results, and more efficient storage, transferring, and archiving data [11, 12]. Since 1986, the Image processing techniques have been applied on cephalometric analysis and landmarks measurements. Several image processing approaches were used to extract the important features of X-Ray images to detect the landmarks for geometrical measurements [13, 14]. Early works were used edge detection technique to locate the landmarks points, and cephalometric classes are then identified by geometrical relations of angles, lines, and intersection and exterior boundaries. Thus, researchers have been focused to develop several systems to automate the analysing and measurements process of cephalometric using several approaches such as resolution pyramid, and Edge enhancement [15], Pattern matching [16], Active shape models [17], Active contours with similarity function [18], PCNN (pulse coupled neural networks) [19], Support vector machines [20], Filtering, Edge tracking, pattern matching, and Active shape models [21].

Current systems have been developed to transfer the traditional process of cephalometric to be performed automatically using digital devices. Research applied image processing in cephalometric field to transfer X-ray films into computing devices to be stored as images for further processing such as enhancing X-ray images, locating landmark points (either automatically or manually), calculating the angular and linear parameters, and following the case status. In more details, X-ray image enhancements is included in most cephalometric systems by applying specific filters to increase image contrasts such as kalman [22], S.Ti.F. [23], unsharp, and Gaussian [24, 25]. Furthermore, cephalometric systems were implemented locating landmark points either manually by allowing specialist to select the interested points in computing screen device, or automatically by allowing system to detect and identify landmarks points using some approaches such as fuzzy logic, and ANN [26, 27]. In addition, some cephalometric systems tried to predict the patient face after surgery [11], while other research tried to develop and evaluate cephalometric system using three dimension devices [28–30]. There are several studies undertaken to compare the accuracy of digital cephalometric with analogue methods [31, 32]. However, some research reported that the manual approaches are still more convenient to the orthodontics than automatic process even though research have shown that the accuracy of some cephalometric system is higher compared with the traditional methods [7, 33]. Research stated that digital methods can be also lead to some errors such as transferring, magnification, and measurements errors. Particularly, existing systems accuracy were varying between 60 and 80% in automating cephalometric compared with manual process, where the total errors should be not more than 0.59 mm for the x coordinate, and 0.56 mm for the y coordinate to be acceptable [34]. Unfortunately, no research on automatic landmark location archives the previous value [35]. Recent study showed that current cephalometric measurements obtained with the computerized cephalometric systems can be considered reliable, and can be used by the clinician [33, 36, 37]. This findings is supported by study perfomed by Paixão et.al [36] which compares between manual and automatic process using Dolphin imaging software on 50 subjects (male and female). The study did not show any significance difference between manual and automatic process [36]. Similar findings have been reported by Tikku et al. using 13 linear and 13 angular measurements on 40 subjects, where only 6 among 13 measurements were significant [37]. However, most studies did not emphasize on the usability aspect of the system. In this research, we aim to develop a cephalometric system, and evaluate its accuracy, performance, and usability against manual process. Usablity is considered as an important aspect of user accaptance of a developed system where the System Usability Scale method (SUS) was applied to indicate the user satisisfication and acceptance level of the develop system.

## Methods

**Fig. 2:**
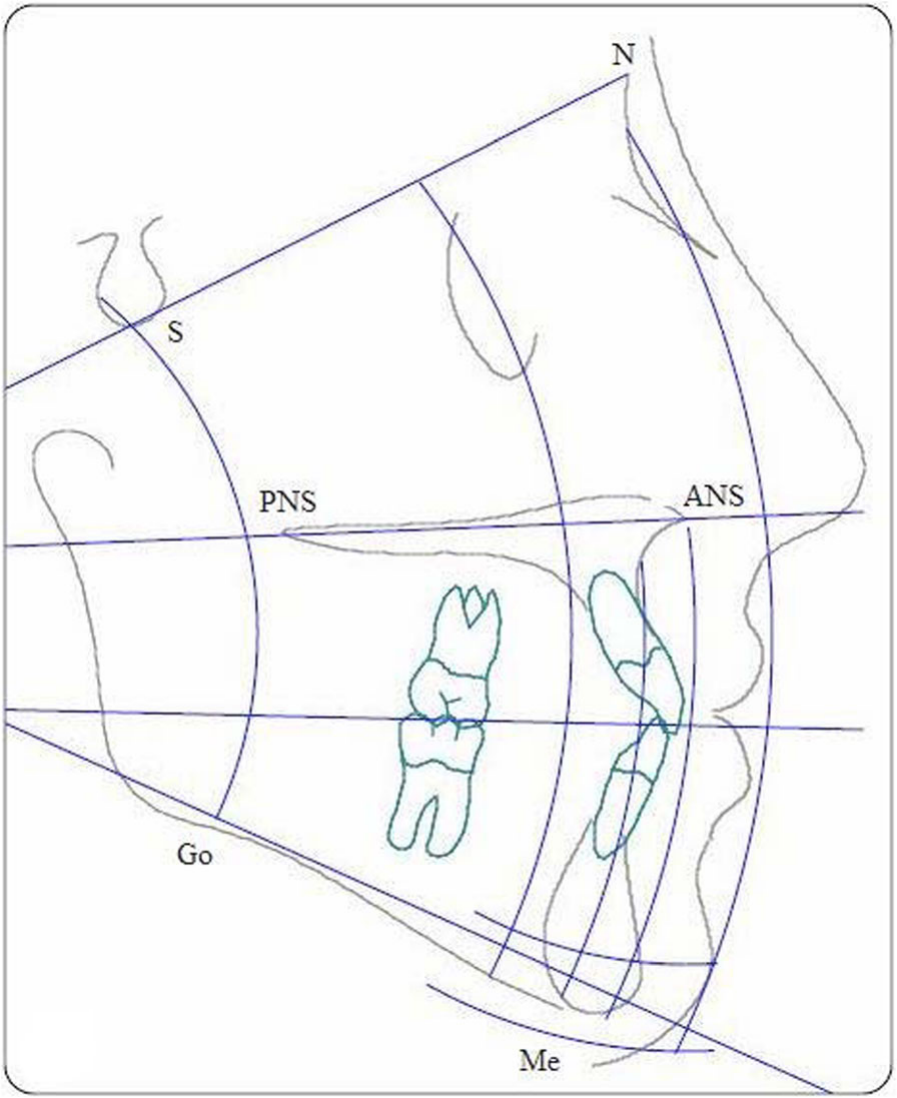
Manual Cephalometric Sample

**Fig. 3:**
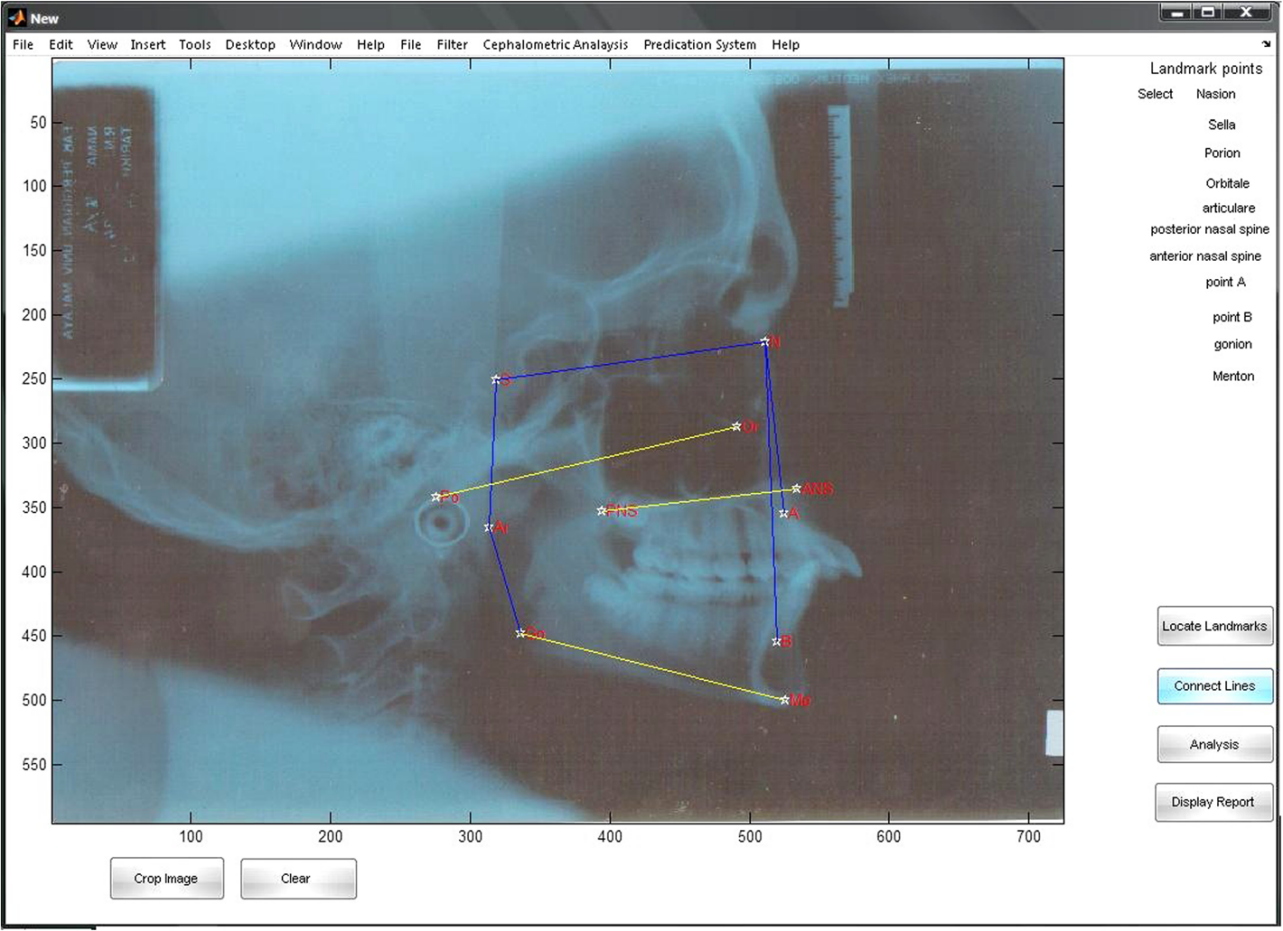
Automatic Cephalometric Sample using Ceph-X

Thirty clinically examined Malaysian adult patients with permanent dentition (up to second molars) with mean age of 21 years old with different ethnics (Malay, Indian, and Chines) were selected in this study. The 30 radiograph samples were obtained with ethical approval from patient archives in the department of orthodontics, University Malaya Hospital. The number of samples used with this study is nearly optimal if compared with similar studies where the differences between the number of samples are around 10–20 samples [36, 37]. Hence the study is retrospective; we hide the patient information to assure the confidentiality, and privacy of patients. Samples were taken by specialist orthodontics and contained the manual tracing for every case. The 30 selected samples converted into digital format and stored in computer with image resolution (1024 × 1024). Matlab 14 software was used to develop the Ceph-X system, and three orthodontics specialists took place in experiments to evaluate the accuracy and performance of Ceph-X. The entire three specialists involved in identifying the 12 common landmarks manually, on both original radiographic films, and on digital images for all samples. They covered the original X-ray film with pellucid papers, and used a pencil to locate landmark points for each case, and landmark identification for the digital images was performed directly on the monitor-displayed image with a mouse-cursor. Geometric tools such as protractor, and ruler were used to construct lines and angles manually through linking the landmark points traced onto the pellucid papers as shown in Fig. 2. Specialists then used The Ceph- X system to perform cephalometric analysis and measurements automatically for each case using the same cephalometric analysis principles as shown in Fig. 3. Furthermore, Ceph-X system usability was evaluated by distributing usability survey designed by using the SUS techniques among 20 orthodontic specialists to get their feedbacks [38]. 11 landmarks points, 12 linear measurements, and 6 angular measurements are used in this study as listed below in Table 1.

**Table 1:** Cephalometric parameters used in this study

Landmark Points (11)	Lines (12)	Angles (6)
N: Nasion	Po - Or	SNA
S: Sella	ANS - PNS	SNB
Po: Porion	Me - Go	ANB
Or: Orbitale	S – N	FMA
Ar: Articulare	N – A	PMP
Go: Gonion	N-B	NSAR
Me: Menton	N-Me	
ANS: Anterior nasal spine	N-ANS	
PNS: Posterior nasal spine	ANS-Me	
Point A: sub spinal	S-Go	
Point B: supramental	S-Ar	
	Ar-Go	

### System development

Ceph-X was developed by applying some image processing techniques to enhance the X-ray images, locate landmark points, and compute automatically linear and angular cephalometric measurements. Four main models were developed, enhancement model, locating model, computing model, and report generation model.

### Enhancement model

**Fig. 4:**
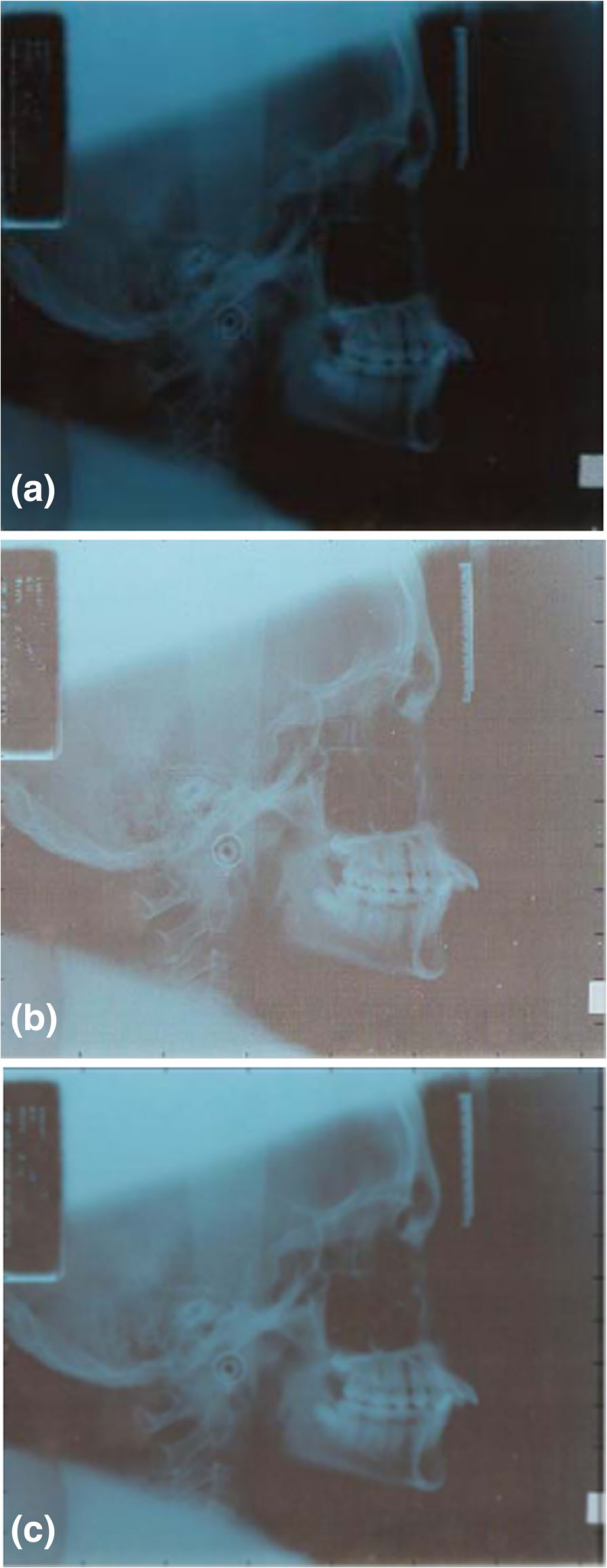
Ceph-X image processing steps. (**a**) Original Image, (**b**) original image after applied Unsharp filter, (**c**) original image after applied Gaussian filter

During X-ray acquisition and transmission process, images are degraded often with several noises which originated from multiple sources. Thus, enhancement X-ray images are necessary to ensure the accuracy of locating and measurement process. Unsharp, and Gaussian filters we were implemented here to enhance the X-ray images because they were reported as the best suitable filters for orthographic image. Results of applying such filters on X-ray images are shown in Fig. 4a, b, c.

### Locating landmark model

**Fig. 5:**
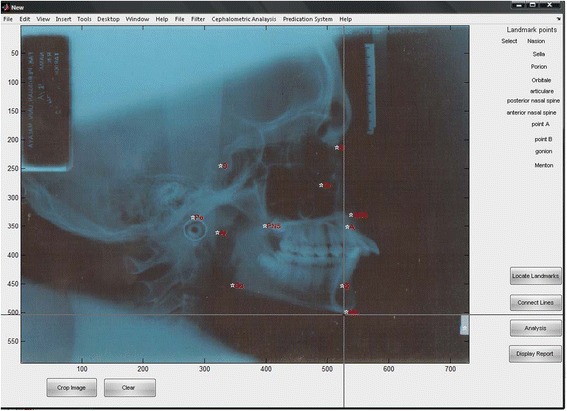
Ceph-X interface during locating landmark points

This model is designed to locate the landmark points manually to reduce the measurement errors stated in previous studies [12]. There are 11 nodes selected to identify the landmark points on the skull, where each node location are registered as a coordinate points of (x, y) for measurement purpose as shown in Fig. 5.

### Measurements model

Measurements model was designed mainly to obtain the measurement results for 18 linear and angular parameters (6 angles, 12 lines) through using some geometrical algorithms as described below.

#### Linear measurements

It developed to connect between specific landmark points, and to calculate the distance between the interest points based on cephalometric principles. It is designed to draw the cephalometric lines over the X-ray images as shown in Fig. 3 above, and to obtain the line measurements in (mm). Landmark points were used as parameters in the image co-ordinate system. The line is represented mathematically between each two landmark points as p1(x1,y1), p2(*x*2,y2) vector. Then, direct path algorithm is used to generate the matrix path points G [Xg, Yg], and line lengths are calculated by using Pythagoras’ equation:

1 $$ {d}^2 = {\left({y}_2-{y}_1\right)}^2+{\left({x}_2-{x}_1\right)}^2 $$

For accurate measurement, we employed two mathematical equations to calculate the resolution of a computer display or image pixel density (PPI), and to make unite conversion between pixel and inch automatically as shown below.

2 $$ \mathrm{P}\mathrm{P}\mathrm{I}\ \left(\mathrm{Pixels}\ \mathrm{per}\ \mathrm{inch}\right) = \mathrm{image}\ \mathrm{resolution}/\mathrm{screen}\ \mathrm{resolution} $$

3 $$ \mathrm{Distance}\ \mathrm{in}\ \mathrm{in}\mathrm{ch} = \mathrm{Distance}\ \mathrm{in}\ \mathrm{pixel}/\left(\mathrm{P}\mathrm{P}\mathrm{I}\right) $$

These equations are used mainly to reduce the measurement errors for linear measurements based on the factor scale.

#### Angular measurements

Designed to calculate specific angles in degree according to cephalometric principles. Theoretically, angle can be formed by connecting any three points, or by intersection of two lines in (X,Y) plan. In the 2 dimension space, we used the following formula to obtain the angle θ between each two lines.

4 $$ \uptheta ={ \tan}^{-1}{\scriptscriptstyle \frac{m1-m2}{\left(1+m1*m2\right)}} $$

where *m1*, and *m2* are the slop of line L1, and line L2 respectively. We used the mathematical equation below to obtain *m1*, and *m2* by finding the changes between each two arbitrary points (X1, Y1) and (*X*2, Y2) of the line.

5 $$ \mathrm{m} = \frac{Y2-Y1}{X2-X1} $$

Then, a conversion process of angles is performed from radian scale into degree scale using the following equation.

6 $$ \mathrm{Angle}\ \left(\mathrm{degree}\right) = \mathrm{Angel}\ \left(\mathrm{radian}\right)/\left(\left(2*\mathrm{pi}\right)/360\right) $$

This conversion process is necessary because orthodontics are more familiar to understand angles in degree.

### Reporting model

The output of Ceph-X is a data file contains angular and linear results, which generated automatically to be displayed for orthodontics usage as html report.

## Results

In this study, two methods have been conducted to evaluate the reliability and usability of Ceph-X, as described in detail below.

### Ceph-X reliability

Reliability evaluation is used to evaluate the accuracy and performance of Ceph-X. Expert orthodontics participated in this evaluation by performing cephalometric measurements using both manual and digital approach. 18 measurements parameters (6 angles and 12 lines) for each case among the 30 case samples were used to evaluate Ceph-X accuracy. These parameters were measured by orthodontic using the manual and automatic approaches. Data have been classified into two groups includes the data of manual procedure, and Ceph-X data. The results of manual and automatic measurement were analysed to obtain the mean values and standard deviations for the linear and angular measurements. Additionally, results of the manual and automatic approaches for both linear and angular measurements were analysed by applying the *t*-test at the significant level of *P* value < 0.05 as shown in Tables 2 and 3. Statistic results of mean and standard division showed slight differences between the automatic and manual data.

**Table 2:** Comparison results for linear measurements

parameter/Case	Automatic		Manual		*T*-test
	Mean	SD	Mean	SD	
Po - Or	7.39	0.534	7.59	0.652	0.01
ANS - PNS	4.66	0.217	4.74	0.25	0.0016
Me - Go	5.72	0.397	5.85	0.497	0.004225
S – N	6.36	0.241	6.44	0.207	0.0016
N - A	4.63	0.309	4.74	0.299	0.003025
N - B	5.74	0.317	5.87	0.333	0.004225
N-Me	10.53	0.678	10.76	0.706	0.013225
N-ANS	4.42	0.257	4.44	0.28	0.0001
ANS-Me	6.4	0.641	6.53	0.432	0.004225
S-Go	7.5	0.365	7.64	0.417	0.0049
S-Ar	3.14	0.384	3.11	0.431	0.000225
Ar-Go	4.71	0.401	4.8	0.371	0.002025

**Table 3:** Comparison results for angular measurements

Parameter/Case	Automatic		Manual		*t* - test
	Mean	SD	Mean	SD	
SNA	90.61	2.22	90.7	2.97	0.002025
SNB	86.56	1	86.6	1.72	0.0004
ANB	4.09	1.75	4.1	1.52	0.000025
F.M.A	33.6	1.41	33.9	1.9	0.0225
PP-MP	32.9	1.89	33.3	1.54	0.04
N.S.Ar	112.5	1.85	112.8	1.22	0.0225

Furthermore, Ceph-X performance is evaluated by calculating the time spent in both procedures for each case. Locating landmarks, cephalometric tracing, and measurements process were used to estimate the time spent by orthodontic for each stage of cephalometric analysis and measurements. Table 4 shows the mean, and standard deviation result of the required time for each procedure of manual and automatic cephalometric. In addition, the maximum errors were obtained between the manual and automatic procedures, which equal 1.15°, and 0.16 mm approximately for angular and linear measurements respectively.

**Table 4:** Comparison results of performance evaluation

Time Parameter	Manual (*N* = 30)	Automatic (*N* = 30)	*P* - Value
Locating Landmarks	1.72 0.26	.62 0.12	*
Cephalometric Tracing	10.45 0.46	0.73 0.1	*
Measurements	14.17 1.52	.2	*

*Significant difference between Manual and automatic groups at *P* <0.05

### Ceph-X Usability

SUS approach is used to evaluate the usability of Ceph-X system. SUS approach abbreviation for (System Usability Scale) is used because it proves its reliability, and validity with approximately more than 2800 citations [38]. 20 novice and expert orthodontics were guided to use Ceph-X for analysis and measurements several cephalometric cases. Then, SUS survey was distributed among them, to gather their opinions about Ceph-X. Result of interpreting the SUS scores from participants indicated an excellent usability scale about Ceph-X system.

## Discussion

This study is conducted to provide a clear picture about the possibility of replacing the traditional cephalometric process with the digital one. The study focused mainly to design a usable cephalometric system, and evaluate its reliability and usability for cephalometric analysis and measurements using SUS method. No differences in gender have been found in this study as it is in accordance to findings stated in literature [39, 40]. Ceph-X obtained a high accuracy results with approximately 96.6% compared with traditional method. Data in Tables 2 and 3 showed that there are no significant differences between the Ceph-X and traditional approach in cephalometric measurements. The maximum error results which approximately 1.15, and 0.16 mm for angles and lines respectively, is still acceptable on cephalometric measurements, in agreement with previous studies and acceptable clinically [9, 34, 36, 37]. High accuracy results of Ceph-X was achieved because of system ability to enhance and zoom the X-ray images, and also because we excluded the automatic landmark locating which considered as one of main errors source of digital conversion for cephalometric process as stated previously [33, 35]. The cephalometric measurements (12 linear and 6 angular) used in this study are selected according to the most important landmarks points. These points are easily identified, uniform in outline and reproducible and permits valid quantitative measurements of lines and angles projected from them [39, 40]. The results of this study shows the statistical differences for linear and angular measurements in digital and manual methods are clinically acceptable based on criteria set by [9, 29]. The findings in this study also conforms to the study conducted by [36] of 50 subjects in terms of cephalometric parameters (6 linear and 8 angular measurements) and mean age. However in this study a single examiner performed manual tracing which can lead to inter examiner error and the reliability of the measurement taken despite of using larger sample of 50 subjects. Inter and intra examiner error is assessment of reliability is important when identifying landmarks measurement in orthodontic studies. In order to avoid intra-examiner error the current study used three orthodontic specialists to obtain the measurements. Mean value of measurement taken by all three of the orthodontics are used in this study to increase the reliability of the study. In addition, result showed that there is no significant difference between the manual and automatic approaches for all the 12 linear and 6 angular parameters used in this study. Study conducted by Tikku et al. [37] using more parameters (13 linear and 13 angular) measurements of 40 subjects indicated that only 6 out of 13 angular measurement used in the study were statistically significant. Therefore it can be concluded that usage of extra angular measurement as reported in [37] leads to complicated system which reduces the system usability. Both studies conducted by Tikku et al. [37] and Paixao et al. [36] have disregarded the usability aspect of the system which have been addressed in the current study. The SUS method have been used to measure user usability and Ceph-X is developed using measurements which are significant and is it sufficient to be used in routine clinical practice.

The mathematical equations implemented in Ceph-X had enhanced the system accuracy by converting the different measurements unites between the digital and manual process, and by obtaining the linear and angular measurements similar with traditional methods. In addition, Table 4 showed that there is significant differences on time between the comparisons of manual and computerize methods in all of the cephalometric analysis and measurements stages. Thus, Ceph-X proved its efficiency in reducing the orthodontics time, and efforts required for cephalometric process, with performance results approximately more than 10 times if compared with the manual approach. Furthermore, an excellent usability result for the Ceph-X showed that orthodontics are ready to replace the traditional cephalometric process with the computerize methods, where usability score result using the SUS method also showed that users preferred using Ceph-X system instead of the manual approach in disagreement with previous research [7, 34]. Thus, efficiency of Ceph-X system in reducing their time and efforts of cephalometric analysis and measurements, and the additional advantages of computer system were behind the Ceph-X user’s satisfactions. Even though the current study is using 30 subjects intra examiner error was taken into consideration to ensure the reliability and SUS method has been applied to ensure the usability of the study as compared with previous studies [36, 37]. Overall, this study proved the possibility of achieving a high reliability results for cephalometric process if conventional approach was replaced with suitable digital approach, in agreement with the finding of several studies [41]. Ceph-X system had a very small error because it was implemented mathematically to resolve the scaling factors errors and conversion process errors during cephalometric measurement. These results in better speed, accuracy and consistency enhance the overall value of the Ceph-X system for the clinical usage.

## Conclusions

This work shows that automatic system for cephalometric analysis and simulation can be achieved if suitable computer system is developed. Ceph-X proved its reliability and usability with clinically acceptable errors to be replaced the manual process for cephalometric measurements. Future studies will be carried out on larger cohort to optimise and eventually increase the land mark point list. Future study will also include study on differences in results obtained based on ethnicity and the possibility to use 3D CT scans.

Ceph-X reduced the time and efforts required for cephalometric process specifically for obtaining cephalometric measurements compared with using the ruler and protractor in manual approach. A cephalometric system supports users with additional digital advantages such as easy storage, archive, access, and transmission patient information, with the ability of image manipulation and processing.

Typical cephalometric system should be included image enhancement, landmarks locating, linear and angular measurements, and report generation models. Automatic landmark locating model should be omitted in cephalometric system because it’s a potential errors source. Ceph-X system is essentially preferred by orthodontics for its reliability, user friendly, and time and effort saving.
